# Antioxidant Bioactive Agents for Neuroprotection Against Perinatal Brain Injury

**DOI:** 10.3390/cells14110818

**Published:** 2025-05-31

**Authors:** Virginia Beretta, Elena Scarpa, Silvia Carloni, Chiara Petrolini, Valentina Dell’Orto, Sebastiano Ravenda, Serafina Perrone

**Affiliations:** 1 Neonatology Unit, Department of Medicine and Surgery, University of Parma, Pietro Barilla Children’s Hospital, 43121 Parma, Italy; virginia.beretta@unipr.it (V.B.); elena.scarpa@ao.pr.it (E.S.); cpetrolini@ao.pr.it (C.P.); valentinagiovanna.dellorto@unipr.it (V.D.); 2 Department of Biomolecular Sciences, University of Urbino Carlo Bo, 61029 Urbino, Italy; silvia.carloni@uniurb.it; 3 Stress Physiology Lab, Department of Chemistry, Life Sciences and Environmental Sustainability, University of Parma, Viale delle Scienze 11, 43125 Parma, Italy; sebastiano.ravenda@unipr.it

**Keywords:** oxidative stress, brain injury, neuroprotection, drugs, newborn infants

## Abstract

Physiological oxidative stress plays a pivotal role in supporting proper growth and development. While moderate oxidative stress is essential for activating key metabolic pathways and maintaining normal cellular signaling, excessive production of reactive oxygen species (ROSs) can overwhelm the immature antioxidant systems of newborns, potentially leading to cellular damage and impaired physiological function. This vulnerability is particularly pronounced in the central nervous system, where limited detoxification capacity exacerbates the risk of oxidative damage, following hypoxic–ischemic events. Antioxidants agents—such as melatonin, erythropoietin, allopurinol, N-acetylcisteine, selenium, iminobiotin, taurine, and acetyl-L-carnitine—have demonstrated significant neuroprotective effects in preclinical experimental studies, reducing markers of oxidative injury and improving neurological outcomes. These neuroprotective agents have also been evaluated in clinical trials, demonstrating antioxidant effects. A major issue lies in the complexity of neurological damage, which is not associated with a single pathological pathway. Additionally, the inability of these agents to reach effective concentrations within the central nervous system, along with inconsistencies across clinical trials in terms of dosage and administration methods, hinders the ability to obtain robust results. Future efforts should therefore focus on the development of delivery systems capable of crossing the blood–brain barrier and on establishing standardized clinical trial protocols and study designs. This educational review aims to provide a comprehensive overview of emerging protective strategies, including antioxidant bioactive agents and nutritional interventions. It also explores the underlying mechanisms of oxidative stress and its impact on neonatal brain injury.

## 1. Introduction

Oxidative stress (OS) refers to the condition in which the production of pro-oxidants overwhelms antioxidant cellular defenses, leading to cellular damage [1]. In biological systems, pro-oxidants are mainly represented by reactive oxygen species (ROS) and reactive nitrogen species (RNS), comprising both free radicals (FRs) and their non-radical intermediates [2]. ROSs consist of free radicals, such as superoxide (●O_2_^−^) and hydroxyl radicals (●OH), as well as non-radical molecules like hydrogen peroxide (H_2_O_2_) and singlet oxygen (^1^O_2_). RNSs comprise nitric oxide (NO●), which is relatively unreactive, and its more reactive derivative, peroxynitrite (ONOO^−^). These species are produced endogenously during cellular metabolism via both enzymatic and non-enzymatic pathways. Enzymatic sources include the mitochondrial respiratory chain, phagocytic oxidative bursts, prostaglandin synthesis, and the cytochrome P450 enzyme system; non-enzymatic generation occurs through the interaction of molecular oxygen with organic substrates or upon exposure to ionizing radiation [3]. FRs are molecules with a single unpaired electron on the outer shell, which confer them the ability to react rapidly with other molecules [4], such as proteins, lipids, or DNA, altering their structure and function. ROSs and RNSs also play an important role in intracellular signaling cascades regulating vascular tone and cell proliferation, differentiation, and migration [5,6].

Under pathological conditions, NO reacts rapidly with superoxide anions to form peroxynitrite, a highly reactive and damaging species. Peroxynitrite causes nitration of tyrosine residues on proteins and generates hydroxyl radicals, which are potent inducers of lipid peroxidation and DNA damage, amplifying oxidative stress and cellular injury.

Additionally, NO impairs mitochondrial respiration by inhibiting critical components of the electron transport chain, notably cytochrome oxidase (complex IV) and complex I. This disruption leads to energy failure and activates apoptotic pathways, further contributing to neuronal loss [7,8]. Thus, excessive NO levels are not only cytotoxic but also interfere with essential mitochondrial function.

The effects of FR are well controlled by antioxidant cellular defenses, which comprise antioxidant enzymes (catalases, glutathione peroxidases, and superoxide dismutases), small molecules (ascorbate, alfa-tocopherol, glutathione, ubiquinone, etc.), and adaptive mechanisms leading to a higher expression of antioxidant genes. This balance may be altered by an increased production of FRs (toxin exposure, reperfusion after hypoxic–ischemic insult, activation of endogenous enzyme in chronic inflammatory disease) or a deficit in antioxidant defense (nutritional deficit, immaturity of enzymatic activity), leading to OS and damage [1,9]. Newborns—especially those born preterm—are exceptionally vulnerable to oxidative stress. This heightened susceptibility stems from several factors: their antioxidant enzyme systems are underdeveloped, their rapidly increasing energy requirements place a heavy burden on aerobic metabolism, and certain conditions elevate free iron levels, which in turn drive excessive free-radical formation [9,10,11]. OS is a key mediator of organ damage in the distressed fetus and neonate and underlies many prematurity-related complications, including bronchopulmonary dysplasia, retinopathy of prematurity, necrotizing enterocolitis, intraventricular hemorrhage, periventricular leukomalacia, and punctate white-matter lesions, which may be part of one entity defined as “the oxygen radical disease of neonatology” [11]. OS is now recognized as an important contributing factor of perinatal disease, including brain injury [9,12].

### Oxidative Stress and Neonatal Brain Injury

**Figure 1:**
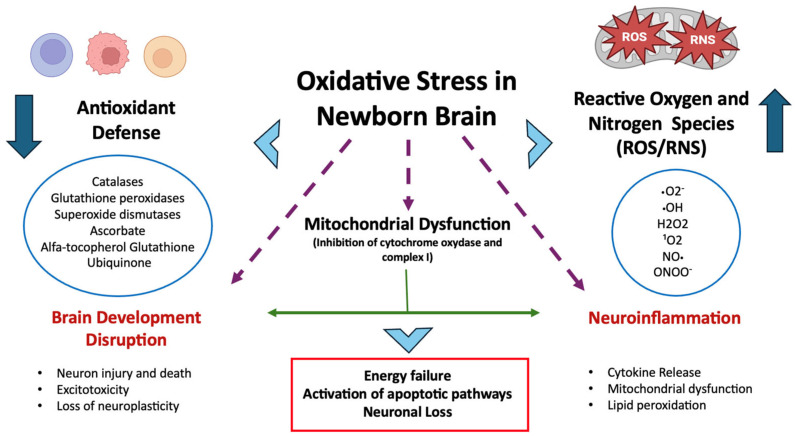
Oxidative stress in the neonatal brain arises from increased ROS/RNS production (e.g., due to hyperoxia, ischemia–reperfusion, inflammation) and immature antioxidant defenses. This imbalance leads to mitochondrial dysfunction, neuroinflammation, impaired brain development, and brain damage.

During the perinatal period, oxidative stress intensifies as a result of inflammation, hyperoxia, hypoxia, ischemia–reperfusion injury, activation of neutrophils and macrophages, and the release of glutamate and free iron [10,11,12,13]. Hypoxia shifts cells toward anaerobic metabolism, causing a rapid buildup of lactic acid and accumulation of reduced intermediates in the mitochondrial electron-transport chain, which in turn drives excessive free-radical generation [11]. Other pathways fueling ROS production and membrane lipid peroxidation include phagocyte activation, arachidonic acid metabolism via cyclooxygenase and lipoxygenase enzymes, reactions catalyzed by elevated intracellular Fe^2+^, and increased xanthine oxidase activity stemming from accelerated ATP breakdown [14,15]. The combination of high free-iron levels and immature iron-binding/metabolizing systems in newborns further exacerbates their vulnerability to oxidative stress [16,17] (see Figure 1).

The brain is particularly vulnerable to free-radical damage because of its very high rate of oxygen consumption, low concentration of antioxidants, water, and high free iron content [13,18]. In addition, neuronal membranes contain a high proportion of polyunsaturated fatty acids (PUFAs), rendering them especially susceptible to oxidative stress, causing devastating brain damage [19]. The newborn brain has large amounts of unsaturated fatty acids, namely adrenic acid, which has its highest level in myelin sheets, and docosahexaenoic acid, which is more present in neuronal membranes as cell bodies and dendrites in gray matter [19,20]. Newborns exhibit low levels of myelination and diminished expression of key ROS-detoxifying enzymes—namely superoxide dismutase, glutathione peroxidase, and catalase—resulting in an underdeveloped antioxidant defense that heightens vulnerability to oxidative injury. In this context, free radicals markedly promote excitotoxicity, neuronal death, and mitochondrial dysfunction [21]. Experimental strategies that boost antioxidant capacity—through overexpression or pharmacological modulation of superoxide dismutases—have demonstrated neuroprotective effects against oxidative insults [13,22]. Additionally, in the immature brain, both nitric-oxide synthase activity and mitochondrial electron leakage are major sources of free radicals [23,24]. OS and inflammation are tightly linked: ROSs can initiate inflammatory pathways, and, conversely, inflammation generates further ROS [25,26]. Finally, oxidative damage undermines blood–brain barrier integrity via multiple mechanisms, including direct injury to endothelial proteins, lipids, and DNA, disruption of tight junctions, activation of matrix metalloproteinases, and cytoskeletal reorganization [15,27].

## 2. Antioxidant/Neuroprotective Strategies in Experimental and Clinical Studies

Hypothermic treatment is the standard of care for moderate to severe hypoxic–ischemic encephalopathy (HIE). The neuroprotective effects of hypothermia are multifactorial and include also the post-depolarization release of excitatory amino acids and the suppression of OS, inflammation, abnormal receptor activity, and cell death pathways [28,29]. Therapeutic hypothermia has been shown to significantly reduce both mortality and major neurodevelopmental disability, with a number-needed-to-treat of seven [30]. In addition, using room air for the resuscitation of hypoxic neonates has been advocated to minimize reoxygenation-induced injury [31,32], and the latest international newborn resuscitation guidelines now recommend air as the initial gas for neonatal resuscitation [33].

As an adjunct to these measures, antioxidant therapies may provide further neuroprotection after perinatal oxidative stress [34]. The main experimental and clinical studies investigating the use of antioxidant bioactive agents for neuroprotection are reported in Table 1 and Table 2. Such interventions can act at multiple stages of the oxidative injury cascade: by directly scavenging reactive oxygen species, by inhibiting further free-radical generation, by modulating endogenous antiradical defenses, by boosting overall antioxidant levels, and by incorporating lipophilic antioxidants into cell membranes to strengthen their resistance to lipid peroxidation [35,36].

**Figure 2:**
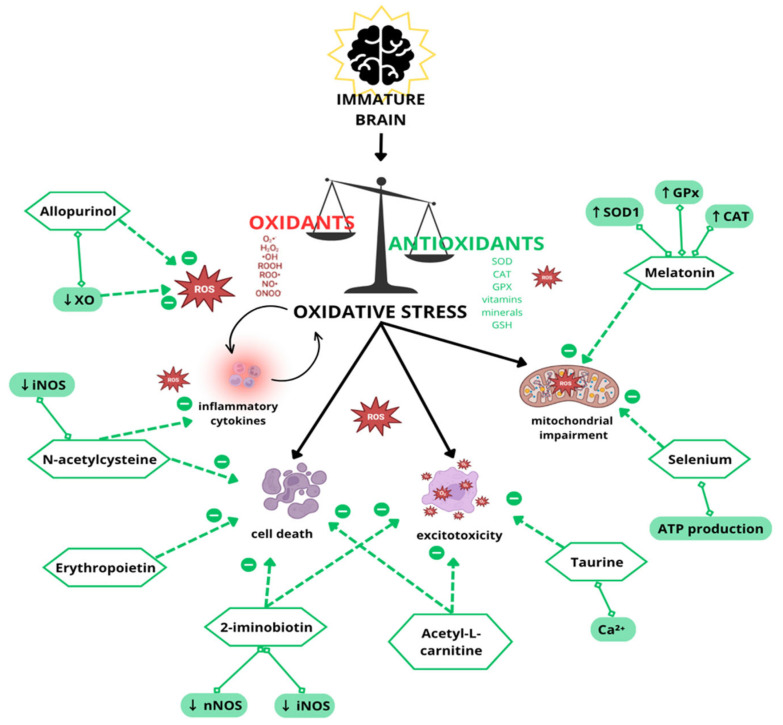
Schematic representation of mechanisms contributing to oxidative stress and brain injury, including excitotoxicity, mitochondrial impairment, and blood–brain barrier breakdown. This figure also highlights potential antioxidant therapies with protective effects.

The mechanisms leading to brain injury and the potential protective effects of antioxidant therapies are illustrated in Figure 2.

### 2.1. Erythropoietin

Erythropoietin (EPO) is a glycoprotein hormone with erythropoietic and non-erythropoietic effect. EPO may exert a neurotrophic effect through the binding with receptors expressed in neurons, astrocytes, and microglia. In particular, EPO may prevent cell death for its antiapoptotic, anti-inflammatory, and antioxidative actions; moreover, it may promote neuroregeneration and angiogenesis [74,75]. Following cerebral hypoxia, Epo is produced by astrocytes, oligodendrocytes, endothelial cells, neurons, and microglia. A specific receptor named EpoR has been isolated in glial cells, neurons, and brain endothelial cells in the hippocampus, cortex internal capsule, and midbrain regions. When Epo binds to EpoR, cellular survival is promoted, with angiogenesis, oligodendrogenesis, and neurogenesis [75,76].

In a full-term nonhuman primate model of perinatal asphyxia, combining therapeutic hypothermia with erythropoietin led to an increased N-acetylaspartate-to-creatine ratio and a decrease in choline levels during the first 72 h after birth [37].

A nonhuman primate study and small clinical trials provide evidence of the safety and efficacy of a multiple-dose EPO regimen when combined with therapeutic hypothermia (TH), suggesting that EPO has been associated with reduced MRI-detected brain injury, better neuromotor outcomes, and a lower incidence of death or moderate-to-severe cerebral palsy in several studies [37,38,39,77]. However, Sheldon et al. reported that EPO fails to mitigate damage when hydrogen peroxide accumulates excessively and may even worsen injury under conditions of extreme oxidative stress [39]. This suggests that EPO may offer little benefit—or could be harmful—if administered immediately following a severe hypoxic–ischemic insult, possibly because exogenous EPO interferes with the brain’s own early repair mechanisms [57].

Conversely, in settings of milder injury, EPO-based therapies might still favorably alter the course of damage. Yet, a large trial of high-dose EPO given to extremely preterm infants from 24 h after birth until 32 weeks postmenstrual age showed no reduction in overall morbidity or mortality [64].

Eight clinical trials have evaluated the neuroprotective effects of erythropoietin in term infants with hypoxic–ischemic encephalopathy, enrolling 533 neonates at 36–42 weeks’ gestation [56,58,59,60,61,62,63,78]. EPO was initiated within the first postnatal weeks at doses ranging from 200 to 2500 U/kg per administration, given either as a single dose or once daily for three to five days. None of these studies reported increased mortality or disability among EPO-treated infants; the therapy was well tolerated, with no allergic reactions, venous thrombosis, or electrolyte, hepatic, or renal function abnormalities observed [56,61]. As expected from its hematopoietic action, EPO-treated infants also exhibited higher hemoglobin levels and red blood cell counts compared with controls [56].

In two studies, EPO was administered in combination with TH and compared to TH alone [62,78]. Trials comparing EPO to placebo or supportive care alone reported a reduction in both mortality [58,59,61] and disability at 18 months of age [58,61]. Additionally, treatment with EPO alone was associated with reduced brain injury on MRI performed between 10 and 14 days of life [61].

The efficacy of EPO compared to TH alone yielded mixed results. One study found that EPO treatment in moderate HIE led to lower rates of mortality and disability, as well as higher scores on the Bayley Scales of Infant Development-II (BSID-II) at 18 months [56]. However, another study reported a less pronounced effect of EPO on mortality and disability compared to TH alone [59].

The combination of EPO and TH was shown to be safe, with no evidence of increased brain injury on MRI between days 4 and 13, and no adverse impact on disability at 22 months [60]. Furthermore, the combined therapy was associated with lower mortality [78], reduced brain injury scores on early MRI (days 4–7) [62,78], and improved neurodevelopmental outcomes at 12 months compared to TH alone [78].

The findings across these studies suggest that EPO is a safe and potentially effective adjunct to TH in managing newborns with HIE. Although results indicate a reduction in mortality, brain injury, and better neurodevelopmental outcomes, some variability in the effects, particularly when compared to TH alone, highlights the need for further research. Standardized protocols and larger randomized controlled trials are necessary to conclusively determine the optimal dosing, administration schedules, and long-term benefits of EPO therapy in this population.

### 2.2. Melatonin

Melatonin, along with several of its metabolites, functions as a potent direct scavenger of free radicals. It also exerts significant indirect antioxidant effects by enhancing mitochondrial electron transport efficiency and stimulating the activity of key antioxidant enzymes, such as superoxide dismutase, catalase, and glutathione peroxidase. Notably, unlike many other antioxidants, melatonin does not exhibit pro-oxidant properties [66,79].

Numerous preclinical studies have demonstrated melatonin’s neuroprotective potential following perinatal hypoxic–ischemic (HI) injury [42,44,46,80]. Its dual action—directly neutralizing ROS and indirectly modulating antioxidant enzyme systems—contributes to mitochondrial protection, supports ATP production, and inhibits oxidative stress-induced apoptosis [42,44,46]. In a newborn piglet model of HI, the combined use of hypothermia and melatonin significantly improved cerebral metabolism in the deep gray matter during the first 48 h after injury, resulting in reduced cellular damage [47].

In preterm and near-term fetal sheep models subjected to intrauterine asphyxia via umbilical cord occlusion, melatonin treatment reduced oxidative stress [40], attenuated cell death, and lessened the neuroinflammatory response [41]. Furthermore, melatonin was shown to decrease caspase-3 activation and improve cellular survival in in vivo models [43]. In juvenile rats, it also improved long-term outcomes, including cognitive and sensorimotor function [45].

More recently, Albertini et al. explored the impact of melatonin on miRNA expressions in a preclinical HI model. They found that HI significantly increased serum levels of miR-126 and miR-146a while decreasing their expression in the ischemic cerebral cortex. Melatonin administration restored both peripheral and cerebral levels of these miRNAs, suggesting a role in modulating ischemic damage at the molecular level [48].

In human studies, melatonin has also shown promise. Its administration to asphyxiated neonates resulted in decreased serum levels of oxidative markers, such as malondialdehyde (MDA) and nitrite/nitrate within the first six hours of life [81]. Aly et al., in a pilot clinical trial, assessed the effect of melatonin in neonates with HIE undergoing whole-body therapeutic hypothermia. Their findings demonstrated that early enteral melatonin administration reduced oxidative stress, improved survival, and was associated with fewer seizures and less white matter damage as seen on MRI and EEG follow-up [65].

Building on this, Weiss et al. conducted a comparative analysis of human and animal specimens to better understand melatonin’s role in neonatal HI injury. A human neonate receiving melatonin and TH was compared to melatonin-treated and untreated HI rats. An analysis of the microRNA expression in plasma and brain tissue helped identify key KEGG pathways associated with HI injury and melatonin response. Expression of proteins such as PKCα, p-Akt, and p-ERK in a rat brain cortex validated a shared, melatonin-sensitive signaling pathway, enhancing translational insight into melatonin’s therapeutic mechanisms [82].

Overall, melatonin’s pleiotropic neuroprotective effects and favorable safety profile make it a promising candidate for adjunctive therapy in neonatal brain injury. However, further research is needed to define the optimal dose, formulation, and timing of administration [66].

### 2.3. Allopurinol

One of the major sources of ROS during hypoxic–ischemic events is xanthine oxidase. In the initial hypoxic phase, as cells switch to anaerobic metabolism, hypoxanthine accumulates. Upon reoxygenation, xanthine oxidase catalyzes the oxidation of hypoxanthine to xanthine and subsequently to uric acid, generating FRs in the process [49,67].

Allopurinol inhibits xanthine oxidase, thereby preventing the conversion of hypoxanthine into xanthine and uric acid and effectively limiting the excessive production of ROS and the associated reperfusion injury [68,83]. Although studies on allopurinol in animal models of HI brain injury are limited, it has demonstrated neuroprotective effects. In particular, when administered 15 min after the induction of HI in 7-day-old Wistar rats at a high dose (135 mg/kg), allopurinol significantly reduced acute brain edema and long-term cerebral damage [50].

A clinical study examining the administration of Allopurinol within the first hours of life following moderate asphyxia demonstrated a significantly lower incidence of severe adverse outcomes in infants treated with allopurinol compared to the control group [67].

The pharmacokinetics and pharmacodynamics of allopurinol and its metabolite, oxypurinol, were analyzed in 46 newborns with HIE as part of the ALBINO study [69].

The ongoing ALBINO trial is a randomized, placebo-controlled, double-blind, multinational parallel group study designed to assess the superiority of allopurinol in term and near-term infants with neonatal HIE. The primary endpoint centers on long-term outcomes, specifically defined as survival with neurodevelopmental impairment, death, or survival without impairment at two years of age [84]. The neuroprotective effects of allopurinol appear to be highly time-dependent, with the greatest efficacy observed when administered very shortly after the hypoxic–ischemic insult. This presents a major challenge in clinical practice, where the precise timing of the hypoxic event is often unknown, and diagnosis or initiation of treatment may be delayed. These factors can limit the window of therapeutic opportunity and potentially reduce the effectiveness of early interventions. Therefore, future clinical protocols should consider strategies for quickly identification of high risk patients and timely administration of neuroprotective agents to maximize their potential benefit.

### 2.4. N-Acetylcysteine

N-acetylcysteine (NAC) is a well-established and powerful thiol-containing antioxidant. It acts both as a direct scavenger of oxygen radicals and as a precursor for glutathione synthesis. NAC contributes to the neutralization of ROS, restoration of intracellular glutathione levels, mitigation of redox imbalance, reduction in apoptotic cell death, and suppression of inflammatory cytokines as well as inducible nitric oxide synthase (iNOS), as demonstrated in an adult rat model of stroke [85,86]. In addition, NAC has been shown to activate the cystine/glutamate antiporter, modulating extracellular glutamate levels. In an animal model of HI, pretreatment with NAC increased levels of HIF-1α protein and its downstream targets, EPO and glucose transporter 3, have been observed in the ipsilateral hemispheres of rodents following hypoxic–ischemic injury. Another study demonstrated that administering NAC 10 min after the onset of reoxygenation, followed by continuous infusion, preserved post-resuscitation amino acid neurochemistry at levels comparable to those in sham-treated piglets. Notably, NAC administration did not disrupt cerebral amino acid balance, thereby maintaining cerebral amino acid homeostasis [87].

A follow-up of a randomized controlled trial involving infants with an extremely low birth weight found that postnatal administration of NAC did not provide neuroprotective benefits [88]. However, when administered to term neonates with HIE on days 5–6 of life, following rewarming after TH treatment, NAC was shown to rapidly restore central nervous system glutathione levels, as measured by magnetic resonance spectroscopy [70]. As with allopurinol, the neuroprotective efficacy of NAC has been shown to depend strongly on the timing of administration. In preclinical studies, early intervention—often within minutes of reoxygenation—is crucial to achieve favorable outcomes. However, translating these findings into clinical settings remains complex due to variability in the timing of birth-related hypoxic–ischemic events. These limitations underscore the importance of developing reliable early biomarkers and streamlined protocols for the prompt initiation of therapy.

### 2.5. Acetyl-L-Carnitine

Acetyl-L-carnitine is an acetylated derivative of L-carnitine, a naturally occurring metabolite that facilitates the transport of fatty acids across mitochondrial membranes for energy production. It helps prevent the excessive cytoplasmic accumulation of free fatty acids and provides acetyl-CoA to support mitochondrial energy metabolism [89,90]. Xu et al. investigated metabolic changes following neonatal hypoxic–ischemic injury in rats and evaluated the neuroprotective effects of acetyl-L-carnitine. They observed significant oxidative and osmotic stress, impaired phosphorylation, and a shift toward anaerobic glycolysis in the ipsilateral hippocampus 24 h post-injury. In contrast, the group treated with acetyl-L-carnitine showed lower lactate levels, preserved total creatine levels at 24 h, and reduced lesion size compared to untreated animals. These findings suggest that early administration of acetyl-L-carnitine after HI may exert neuroprotective effects by serving as an energy substrate, enhancing oxidative energy production, and reducing reliance on anaerobic glycolysis [51].

### 2.6. Selenium

Selenium is a vital component of more than two dozen selenoproteins and plays an essential role in antioxidant defense, particularly in neonates [91]. It is crucial for protecting the central nervous system from oxidative damage and for supporting various physiological processes, including infection response, reproduction, thyroid hormone metabolism, and DNA synthesis [92].

El-Mazary et al. found that neonates with HIE had significantly lower serum selenium levels compared to healthy neonates, with the lowest levels observed in those with severe HIE. Moreover, there were significant negative correlations between serum selenium levels and the severity of HIE [71]. These findings suggest that selenium supplementation may help alleviate hypoxic–ischemic-induced neuronal death both in vitro and in vivo. Selenium appears to protect against glutamate and hypoxia-induced cell death by reducing ROS production and increasing the activity of antioxidant enzymes [93,94]. Additionally, selenium helps regulate ATP production and the activity of mitochondrial respiratory chain complexes [52,95], and it can inhibit mitochondria-initiated cell death pathways and autophagy activation, thus promoting neuronal survival. Therefore, selenium supplementation could potentially serve as a beneficial adjunct to hypothermia therapy in treating neonates with HIE in the future.

### 2.7. Taurine

Taurine is a ubiquitous sulfur-containing amino acid that has been suggested to exert neuroprotective effects, particularly in the developing brain. Its concentration is notably high in the immature brain and gradually decreases with age, indicating a potential role in neurodevelopment and protection during early life stages [96,97]. Its synthesis is tightly coupled with GSH, as they share the same precursor, cysteine [97]. Lima and coworkers have found to be decreased after HI in ipsilateral hippocampus at 24 h after HI in rats; this may reflect a decrease in precursor cysteine levels and might contribute to OS in the damaged hippocampus [98]. The concentration of taurine has been found to be significantly elevated in the cerebrospinal fluid of asphyxiated infants, with levels correlating with the severity of hypoxic–ischemic encephalopathy (HIE) and with different clinical outcomes [72,99]. This suggests that taurine may serve as a potential biomarker for the extent of brain injury and recovery prognosis.

Taurine has demonstrated protective effects against glutamate-induced excitotoxicity, which is a key mechanism of neuronal damage following hypoxic–ischemic insults. These neuroprotective actions are mediated through several mechanisms, including modulation of calcium (Ca^2+^) and chloride (Cl^−^) channels, as well as interaction with N-methyl-D-aspartate (NMDA) receptors [53,100,101,102]. Through these pathways, taurine can help stabilize intracellular ion homeostasis, reduce calcium overload, and limit the downstream effects of excitotoxicity, potentially mitigating neural damage in neonates affected by HIE.

### 2.8. Iminobiotin

The activation of NOS and the resulting overproduction of NO are key contributors to HI-induced cerebral injury in the perinatal period.

Inhibiting NOS activity has been proposed as a potential neuroprotective strategy. Specifically, iminobiotin—a selective inhibitor of both neuronal NOS (nNOS) and inducible NOS (iNOS)—has shown promise in experimental studies.

Importantly, iminobiotin does not significantly inhibit endothelial NOS (eNOS), which is responsible for the vasodilatory effects of nitric oxide essential for maintaining vascular tone and cerebral perfusion. This selective inhibition profile represents a critical therapeutic advantage, allowing for the mitigation of neurotoxic NO production without impairing vascular homeostasis. Moreover, iNOS inhibition may exert indirect antioxidant effects by reducing the formation of peroxynitrite (ONOO^−^), a highly reactive and damaging oxidant generated by the reaction between NO and superoxide anion. This contributes to the overall protective effect of iminobiotin against oxidative stress-induced neuronal injury [103,104].

By selectively blocking the excessive production of NO during and after HI, iminobiotin may help to interrupt the deleterious cycle of oxidative damage and mitochondrial dysfunction, preserving neuronal integrity [105].

The pathophysiological link between elevated NO concentrations and increased intracellular Ca^2+^ underscores the importance of NO in signal transduction and cell fate decisions during cerebral ischemia [106]. While basal NO production has physiological roles, excessive NO becomes neurotoxic, promoting both apoptotic and necrotic cell death pathways [107]. These findings support the rationale for exploring NOS inhibitors like iminobiotin in neonatal neuroprotection.

The constitutive NOS isoforms—nNOS and endothelial NOS (eNOS)—produce low physiological levels of NO, which play critical roles in cell signaling and vascular regulation. In contrast, the iNOS is activated under inflammatory or stress conditions, resulting in high-output NO production. This surge contributes significantly to indirect biological effects, such as protein nitrosylation and oxidative damage, exacerbating cellular injury [108]. The pathological activation of nNOS and iNOS following HI events is closely linked to mitochondrial dysfunction and the initiation of apoptotic pathways [109].

2-Iminobiotin, a selective inhibitor of both nNOS and iNOS, has demonstrated promising neuroprotective properties in preclinical models. It effectively interrupts the cytochrome c-caspase 3 apoptotic cascade, providing both short- and long-term neuroprotection in neonatal female rats following HI insult [54]. In neonatal piglets subjected to moderate-to-severe HI injury, a dose of 0.2 mg/kg was shown to be the most effective. Treated animals exhibited significantly reduced protein tyrosine nitration in vulnerable brain regions, such as the thalamus, parietal cortex, and temporal cortex, alongside improved survival and normalization of EEG amplitude within 48 h [55].

Early clinical evaluation includes a Phase IIa trial conducted in Congo, where neonates with birth asphyxia received six doses of 2-iminobiotin (0.16 mg/kg) within 6 h of life, spaced every 4 h. This trial found the treatment to be safe, with no adverse events directly linked to the drug, although it underscored the necessity of adjusting dosing strategies for future trials [73].

Further pharmacokinetic studies in neonates undergoing TH after perinatal asphyxia confirmed that 2-iminobiotin is well tolerated when co-administered with TH. Target plasma concentrations were attained with a regimen of eight doses of 0.08 mg/kg given every 6 h. Importantly, the drug did not produce safety concerns in the short term [110]. However, further clinical trials are needed to determine the optimal dosing regimen, treatment duration, and long-term efficacy of 2-iminobiotin as a neuroprotective adjunct to TH.

## 3. Conclusions

Numerous studies have highlighted the significant therapeutic potential of antioxidants as bioactive agents in mitigating brain damage in newborns. Compounds such as selective inhibitors of neuronal and inducible NOS, allopurinol, melatonin, and EPO have been the subject of extensive investigation in clinical trials. When used as adjunct therapies in combination with hypothermia, these agents have demonstrated beneficial effects against HIE. These agents have shown promising anti-inflammatory, anti-apoptotic, and neuroprotective properties in both preclinical and early clinical studies. This growing body of research underscores the promising role these compounds may play in reshaping the landscape of neonatal neuroprotection.

However, despite encouraging results in animal models, clinical translation remains limited, and several compounds have yet to demonstrate consistent efficacy in clinical trials. A major limitation is the poor bioavailability of these drugs and their restricted ability to cross the blood–brain barrier (BBB), which hinders the achievement of therapeutic concentrations in brain tissue. This barrier represents a critical challenge in neonatal neuroprotection and may partly explain the gap between preclinical promise and clinical outcomes. To overcome this obstacle, various strategies are being explored, including the use of nanocarriers such as dendrimers [111]. These delivery systems remain largely at the preclinical stage and are not yet approved for clinical use in humans. For example, a dendrimer conjugated with N-acetylcysteine (NAC) demonstrated a 10- to 100-fold increase in efficacy compared to free NAC in a rabbit model of cerebral palsy, with notable improvements in neuronal injury, myelination, oxidative stress, and inflammation [111,112]. In conclusion, while antioxidant therapies offer a promising avenue for enhancing neonatal neuroprotection, their clinical application requires further validation. Future research should focus on optimizing drug formulations, improving BBB permeability, and identifying the most effective dosage regimens in combination with therapeutic hypothermia to maximize neuroprotective outcomes in newborns.

## Figures and Tables

**Table 1:** Main experimental studies that have used bioactive antioxidant agents for neuroprotection.

	Reference	Target Population	Intervention	Outcomes	Findings
***Erythropoietin (EPO)***	Traudt et al., 2013 [37]	35 Macaca nemestrina with UCO. GA: 168 ± 1 days.	Groups: (a) saline; (b) EPO only; (c) TH only; (d) TH + EPO; (e) ctrl. EPO: 3500 U/kg × 1 dose i.v., followed by 2500 U/kg × 3 doses (30 min, 24 h, 48 h, 7 d) or 1000 U/kg/day i.v. × 4 doses (30 min, 24 h, 48 h, 7 d).	Blood samples; Behavioral and motor assessment; MRI.	TH combined with 4 doses of EPO: ↓ risk of death or moderate-severe CP to 0%; Normal motor functions; Repeated 1000 U/kg i.v. EPO is a safe and effective dose.
	Wang et al., 2004 [38]	28 Male Wistar rats with MCAo.	rhEPO, i.p., 5000/10,000 units/kg daily for 7 days starting 24 h after MCAo.	VEGF and BDNF. Behavioral Tests. Infarct volume from 7 H&E-stained coronal sections.	rhEPO treatment: ↑ neurological outcome. ↑ VEGF that mediates rhEPO-induced angiogenesis. ↑BDNF levels.
	Sheldon et al., 2016 [39]	10 transgenic mice overexpressing hSOD1-tg; 13 WT littermates (C57/Bl6) and 3 sham mice. PND9.	HI mouse model; Recombinant EPO (5 IU/g, R&D Systems) or vehicle (saline) was administered immediately, at 24 h, and 5 days after HI.	Histological analysis.	↑ injury in SOD-tg mice than WT; No improvement from EPO treatment.
***Melatonin***	Miller et al., 2005 [40]	15 pregnant ewes with singleton fetuses; GA: 124–127 d.	10 min UCO; Maternal melatonin administered i.v. (1 mg bolus + 1 mg/h for 2 h).	Detection of •OH; Fetal and maternal blood sampling for melatonin, blood gases, and PG; Histological analysis of OS and DNA damage.	Maternal melatonin infusion lead to ↓ •OH in the fetal brain; ↓ lipid peroxidation.
	Welin et al., 2007 [41]	15 pregnant Gotland sheep ewes. GA: 89–90	Maternal aseptic surgery; Fetal administration of melatonin, i.v., 20 mg/kg/h for 6 h starting 10 min after reperfusion (n = 9); vehicle (n = 10).	FMAP and FHR; 8-Isoprostane; total and free thiol levels; Histological and neuropathological analysis.	Mel: ↓ 8-isoprostane levels at 6 h; ↓ microglia count and density in several brain regions.
	Carloni et al., 2008 [42]	18 pregnant Sprague Dawley rats. PND7	Groups: (n = 8): (a) HI-Mel 5 mg/kg-PRE; (b) HI-Mel 15 mg/kg-PRE; (c) HI-Mel 5 mg/kg × 3; (d) HI-Mel 15 mg/kg-POST; (e) HI group (n = 10) saline. Mel: diluted in saline 5% DMSO an i.p. injected.	Short- and long-term histological analysis and long-term behavioral assessment.	↓ brain injury in a dose-dependent manner: 5 mg/kg pre-HI (−29%), 3 × 5 mg/kg (−45%), and 15 mg/kg pre-HI (−64%).
	Hutton et al., 2009 [43]	31 pregnant spiny mice. GA: 29 days.	Groups: (a) saline + C-section (n = 4); (b) saline + birth asphyxia (n = 3); (c) Mel + C-section (n = 7); (d) Mel + birth asphyxia (n = 5). Subscapular implantation of osmotic minipump delivering Mel (0.1 mg/kg/day) or saline.	Mel concentrations; Immunohistochemical Staining and Analysis.	Mel treatment: ↓ asphyxia-related cell death in cortical gray matter and the corpus callosum. ↓ inflammatory cells in the cortical gray matter, dentate gyrus, and corpus callosum.
	Signorini et al., 2009 [44]	9 neonatal Sprague Dawley rats. PND 7	Groups: (a) HI; (b) HI + Mel; (c) sham controls. Mel: i.p., 15 mg/kg, single dose 30 min before HI (5% DMSO/saline).	Histological Assessment; Image analysis for intact brain volume; Biochemical Markers; Free iron levels.	HI-Mel: ↓ OS, F2-IsoPs, F4-NeuroPs, and DFO-chelatable free iron in the cerebral cortex; ↓ DHA oxidation; ↓lipid peroxidation.
	Lekic et al., 2011 [45]	40 Timed pregnant Sprague Dawley rats. PND7	Groups (n = 8): (a) sham-naïve; (b) needle-control; (c) GMH (collagenase-infusion); (d) GMH + 5 mg/kg melatonin i.p.; (e) GMH + 10 mg/kg melatonin i.p.	Cognitive function; sensorimotor ability; Cerebral, cardiac and splenic growths.	Systemic Mel treatment: ↓ long-term brain atrophy; near-normal levels of sensorimotor and cognitive function.
	Balduini et al., 2012 [46]	56 Sprague Dawley rat pups. PND7	Groups: (a) HI-Mel: melatonin i.p., 15 mg/kg, 5 min after HI (5% DMSO/saline). (b) V-HI: vehicle 5% DMSO in saline.	Lipid peroxidation; Western blot analyses; Immunohistochemistry.	HI-Mel: ↓ OS, IsoPs, NPs, and NFs after HI injury. ↓ inflammatory cell recruitment and glial cell activation. ↓ED1-positive cells. ↓ GFAP expression in the brain.
	Robertson et al., 2012 [47]	17 male piglets. Age: >24 h.	Groups: (a) HT (n = 8); (b) TH + Mel (n = 9): melatonin i.v. 5 mg/kg over 6 h, starting 10 min after HI and repeated at 24 h.	Cerebral energetics and metabolite ratios; aEEG; Seizure activity; Histology and Biochemical markers; Plasma Mel levels.	TH + Mel: ↑ cerebral energy metabolism. ↑ ATP levels. ↓ cell death in several brain regions. ↓ microglial activation. ↓ proinflammatory markers.
	Albertini et al., 2023 [48]	54 pup rats. PND7	Groups (n = 18): (a) HI + Mel: melatonin, i.p. to 5 min after HI at the dose of 15 mg/kg; (b) CTRL: Sham-operated controls; (c) HI-injured animals.	Serum and brain samples; Quantitative Real-Time PCR.	Dysregulation of miR-126 and miR-146a in neonatal rats in the early phase of HI injury and restored effects after Mel treatment.
***Allopurinol***	Palmer et al., 1990 [49]	63 Wistar rat pups PND7	Right hemisphere HI insult. Randomized to: (a) s.c. injection of allopurinol (0.2 mL); (b) s.c. injection normal saline (0.2 mL)	Water content; Gross neuropathology; Histopathology.	In allopurinol group: ↓ brain water content in the right hemisphere; ↓brain damage;
	Palmer et al., 1993 [50]	65 Wistar rat pups PND7	Right hemisphere HI insult. Randomized to: (a) single s.c. injection of allopurinol 135 mg/kg; (b) equal volume s.c. injection (0.01 mL/g animal weight) of saline.	Morphologic analysis of the brain; Water content; Gross neuropathology; Histopathology.	In allopurinol group: ↓ Acute brain edema; ↓ HI long term brain damage.
***Acetyl-L-carnitine***	Xu, et al., 2015 [51]	12 Sprague Dawley rats. PND7	Randomized to: (a) Control; (b) HI; (c) HI + ALCAR; 4 doses 100 mg/kg immedi-ately after HI, at 4 h, 24 h, and 48 h.	Lesion Characteristics on T2 -Weighted MRI; Metabolic Changes in Hippocampus and Cortex.	↓ lactate (Lac) levels in the ipsilateral hippocampus of HI + ALCAR vs. HI rats at 24 h postinjury.
***Selenium***	Mehta et al., 2012 [52]	43 male C57BL/6J mice.	Sodium selenite, i.p., 0.2 mg/kg for 7 days before ischemia; MCAO for 1 h, followed by 5 or 24 h reperfusion.	Infarct volume, neurodegeneration, oxidative DNA damage, and protein expression.	Selenium administration: ↓ ischemia-induced brain damage; limiting the activation of autophagy; ↓ levels of Beclin 1 and LC3-II. ↓ mitochondrial fragmentation.
***Taurine***	Chan et al., 2014 [53]	Male Sprague Dawley rats. Age: 4–6 weeks.	No intervention.	Field potential recordings; Neurochemistry;	Inhibition of the NMDA receptor complex by taurine.
***Iminobiotin***	Nijboer et al., 2007 [54]	Timed Wistar rats pups.	s.c. 2-IB (10 mg/kg) or vehicle (10 mL/kg) at 0, 12, and 24 h post-hypoxia (Rice-Vannucci);	Histology; Nitrite and Nitrate measurements; Western blot.	2-IB prevent the increase in cytosolic cytochrome c and activation of caspase 3 only in females.
	Bjorkman et al., 2013 [55]	HI insult in 47 term newborn piglets.	Groups: (a): vehicle; (b) 2-IB (0.1 mg/kg, 0.2 mg/kg, or 1.0 mg/kg)	Tissue analyzed for caspase-3 activity, tyrosine nitration, and histology.	Greater survival with a normal aEEG at 48 h and ↓ tyrosine nitration in 2-IB treated group.

Abbreviations: ALCAR: acetyl-L-carnitine; C-section: Cesarean section delivery; BDNF: brain-derived neurotrophic factor; CP: cerebral palsy; DHA: docosahexaenoic acid; DFO: deferoxamine; DMSO: dissolved in dimethyl sulfoxide; EPO: erythropoietin, rhEPO: recombinant human erythropoietin; FMAP: fetal mean arterial blood pressure; F2-IsoPs: F2-isoprostanes; GFAP: glial fibrillary acidic protein; GMH: germinal matrix hemorrhage; i.p.: intraperitoneally; IsoPs: isoprostanes; i.v.: intravenous; H&E: hematoxylin and eosin; HI: hypoxia–ischemia; hSOD1-tg: g human SOD1; MCAO: middle cerebral artery occlusion; Mel: melatonin; NMDA: N-methyl-D-aspartic acid; NFs: neuroprostanes; NPs: neurofurans; OS: oxidative stress; PA: perinatal asphyxia; PG: prostaglandin; PND: postnatal day; TH: therapeutic hypothermia; UCO: umbilical cord occlusion; V-HI: vehicle-treated hypoxic–ischemic; VEGF: vascular endothelial growth factor; WT: wild-type; 2-IB: 2-Iminobiotin; •OH: hydroxyl radical.

**Table 2:** Main clinical studies that have used bioactive antioxidant agents for neuroprotection.

	Reference	Target Population	Intervention	Outcomes	Findings
***Erythropoietin (EPO)***	Zhu et al., 2009 [56]	167 term infants with perinatal HIE. GA: 37 weeks.	Groups: (a) EPO (n = 83); (b) CTRL (n = 84). rhEPO, 300/500 U/kg: 1st dose s.c., then i.v. for 2 weeks.	Blood and CSF sampled pre-dose and at 3 h, 8 h, and 24 h after. Daily neuro exam × 7 days. Follow-up every 6 m until 18 m.	EPO: ↑ neurologic outcomes, for patients with moderate but not severe HIE.
	Wu et al., 2012 [57]	24 newborns with HIE. GA: ≥36 weeks	6 EPO doses i.v.: 250 (n = 3), 500 (n = 6), 1000 (n = 7), 2500 (n = 8) U/kg. Standard HT.	Pharmacokinetic Analysis in plasma and CSF. Laboratory data at 1, 3, 5, and 14 days.	EPO 1000 U/kg per dose intravenously given in conjunction with hypothermia is well tolerated.
	Avasiloaiei et al., 2013 [58]	67 term neonates with PA.	Randomized to: (a) supportive care; (b) PB: 40 mg/kg i.v. within 4 h of birth + supportive treatment; (c) EPO: 1000 UI/kg/day s.c. for 3 days + supportive treatment.	Total antioxidant TAS and MDA. Samples at 4, 24, 48, 72 h, 7 d. Neuro exam at birth and periodically, aEEG.	↑ TAS in EPO; ↓ MDA in EPO; ↓ incidence of sequelae in PB and EPO.
	El Shimi et al., 2014 [59]	45 full-term neonates. GA: ≥36 weeks.	Randomized: (a) no- intervention (n = 10); (b) HT (n = 10); (c) rEPO (n = 10; 1500 U/kg s.c. on day 1); (d) CTRL (n = 15 healthy, GA/sex-matched).	Brain MRI at 21–28 days. Neuro exam at 3 m + short-term neurodevelopmental outcome.	↑ survival in whole body HT; Better MRI and NMS scores at 3 mo in HT vs. rEPO.
	Rogers et al., 2014 [60]	24 newborns with HIE. GA: ≥37 weeks.	EPO doses i.v.: 250 (n = 3), 500 (n = 6), 1000 (n = 7), 2500 U/kg (n = 8). Standard 72 h HT (whole body: n = 21, head: n = 3).	MRI after HT. Neurodevelopmental follow up at 24 months.	↓ rate of death or moderate to severe disability. High-dose Epo also appears to be safe.
	Malla et al., 2017 [61]	100 neonates with HIE. GA: ≥37 weeks.	Randomized: (a) EPO: 5 doses EPO 500 U/kg i.v. every 48 h starting within 6 h of birth; (b) Placebo: 2 mL saline.	Clinical and neurodevelopmental outcomes at 19 m.	↓ death or mod-severe encephalopathy. ↓NO, ↑antioxidants, ↓ glutamate toxicity, ↓ lipid peroxidation, ↓ inflammation.
	Mulkey et al., 2017 [62]	50 term newborns with moderate to severe HIE. GA: ≥37 weeks.	Randomized: (a) EPO: EPO 1000 U/kg; (b) placebo: saline. Standard HT.	Brain MRI post-hypothermia. Development and motor assessment at 12 months.	↓ acute brain injury volume in EPO vs. placebo. ↑ injury volume linked to worse 12-mo outcomes in placebo.
	Lv et al., 2017 [63]	41 neonates with moderate/severe HIE. GA: mean 39 weeks	Randomized: (a): Control (HT alone, n = 20); (b): EPO (rhEPO + HT, n = 21) rhEPO 200 U/kg i.v., in 10% glucose, once daily × 10 days.	Serum tau protein measurement. Neonatal behavioral neurological assessment. Neurodevelopmental outcomes.	↓ Serum tau protein at 8 and 12 days in EPO vs. Control. ↑ NBNA at 7, 14, and 28 days in EPO vs. Control.
	Juul et al., 2020 [64]	941 preterm infants. GA: >24 + 0 and <27 + 6	Randomized: (a) EPO 1000 U/kg IV q48h × 6 doses + 400 U/kg SC 3×/week until 32 + 6 weeks PMA (n = 477); (b) placebo (n = 464).	Head ultrasound on days 7–9 and at 37 weeks PMA. Outcome at 22–26 mo PMA. MRI at 36 PMA.	No reduction in death risk or improved neurodevelopment with high-dose EPO in extremely preterm infants vs. placebo.
***Melatonin***	Aly et al., 2015 [65]	45 newborns. GA: 38 to 42 weeks.	Randomized: (a) HIE − HT (n = 15); (b) Mel + HT (n = 15), 5 daily oral doses of Mel 10 mg kg^−1^; (c): Healthy control (n = 15).	Laboratory evaluations; EEG; MRI; Neurologic and developmental outcomes;	↓ SOD and NO from baseline to follow-up in Mel + HT. ↓ seizure activity in Mel + HT. ↑ survival without abnormalities at 6 months in Mel + HT.
	Marseglia et al., 2021 [66]	36 preterm newborns GA: <37 weeks.	Randomized: (a): MEL group, 0.5 mg/kg/day orally, first week (n = 21); (b) placebo, 0.5 mL 5% glucose (n = 15).	Melatonin and OS biomarker (AOPP, F2-IsoPs, and NPBI) concentrations.	↓ F2-Isopr at 48 h in MEL vs. placebo. No significant effect on NPBI or AOPP at 24/48 h.
***Allopurinol***	Russell et al., 1995 [67]	400 babies GA: 24–32 weeks.	Randomized: (a) allopurinol (20 mg/kg/day via gastric tube × 7 days); (b) placebo.	Adverse outcome; Biochemical Analysis.	Treated patients: Inhibition of xanthine oxidase; ↓ significant in uric acid; ↑ hypoxanthine;
	Kaandorp et al., 2015 [68]	222 pregnant women. GA: ≥36 weeks	Randomized: (a) Single antenatal i.v. dose of 500 mg allopurinol; (b) antenatal i.v. placebo.	Biochemical markers in cord blood; Neonatal outcome;	↓ S100β and neuroketal values in the newborn female of treatment group.
	Chu et al., 2022 [69]	46 term and near-term infants with severe PA from the ALBINO study. GA: ≥36 weeks.	Allopurinol i.v. 20 mg/kg first dose to all; second dose of i.v. 20 mg/kg in 13 and 10 mg/kg in other 13; TH (n = 13)	PK/PD modeling.	In the final PK/PD model, the combined allopurinol and oxypurinol concentration at the half maximal XO inhibition was 0.36 mg/L (95% CI 0.31–0.42).
***N-Acetylcysteine***	Moss et al., 2018 [70]	24 term neonates with HIE after HT. age: 5–6 day.	Neonates received daily i.v. infusions of NAC (25–40 mg/kg every 12 h) and calcitriol (0.03–0.1 mg/kg/day) from 6 h of life until day 10 or discharge.	Magnetic resonance imaging (MRI); Magnetic resonance spectroscopy (MRS); Quantification of GSH.	↑ GSH in basal ganglia within 12–30 min after NAC infusion; No difference in GSH changes between the NAC only (n = 9) and NAC plus calcitriol (n = 14) groups.
***Selenium***	El-Mazary et al., 2015 [71]	60 full term neonates with HIE; 20 healthy term. GA: ≥37 weeks.	No intervention.	Serum selenium levels, clinical chemistry values for early assessment of neonatal status.	↓ serum selenium levels in HIE vs. Controls; Selenium levels showed a negative correlation with HIE severity based on the Sarnat and Sarnat staging system.
***Taurine***	Gücüyener et al., 1999 [72]	22 infants (10 term, 12 preterm) with PA; 10 Ctrl. GA: 30–39 weeks.	No intervention.	Measurement of amino acids in CSF using high-performance liquid chromatography.	↑ ASP, GLU, and TAU levels in CSF of PA vs. Ctrl.
***Iminobiotin***	Biselele et al., 2020 [73]	7 near-term neonates treated with HIE. GA: ≥36 weeks.	6 i.v. infusions of 2-IB (0.16 mg/kg) every 4 h via umbilical catheter (0.75 mg/mL solution); first dose within 6 h after birth.	Pharmacokinetic analysis.	No adverse effects that could be attributed to the use of 2-IB.

Abbreviations: AOPP: advanced oxidation protein products; ASP: aspartate; CSF: cerebrospinal fluid; EPO: erythropoietin; rhEPO: recombinant human erythropoietin; F2-IsoPs: F2-isoprostanes; GA: gestational age; GLU: glutamate; GSH: glutathione; HIE: hypoxic–ischemic encephalopathy; HT: therapeutic hypothermia; i.p.: intraperitoneally; i.v.: intravenous; MDA: malondialdehyde; Mel: melatonin; NAC: N-acetylcysteine; NBNA: neonatal behavioral neurological assessment; NPBI: non-protein-bound iron; PA: perinatal asphyxia; PB: phenobarbital; PMA: post-menstrual age; PK/PD: pharmacokinetics/pharmacodynamics; s.c.: subcutaneous; TAS: total antioxidant status; TAU: taurine; XO: xanthine oxidase.
